# Feasibility and Acceptability of a Mobile Game to Support Smoking Cessation: Repeated Measures Study

**DOI:** 10.2196/54684

**Published:** 2024-08-21

**Authors:** Shelby Goodwin, Jessica A Nastasi, Schyler T Newman, Darion Rapoza, Bethany R Raiff

**Affiliations:** 1 Department of Psychology College of Science and Mathematics Rowan University Glassboro, NJ United States; 2 Department of Psychology University of Florida Gainesville, FL United States; 3 Department of Psychology University of Missouri St. Louis, MO United States; 4 Division of Cardiology Department of Medicine Duke University School of Medicine Durham, NC United States; 5 Entertainment Science, Inc Durham, NC United States

**Keywords:** mHealth, mobile health, smoking cessation, video game intervention, mobile phone

## Abstract

**Background:**

Approximately half of smokers attempt to quit, but 90% of these attempts fail. Video game–based interventions have the potential to address common barriers to evidence-based smoking cessation treatment, including high cost, lack of health care access, and low engagement.

**Objective:**

The purpose of this study was to evaluate the feasibility and acceptability of a video game–based smoking cessation intervention called Inspired and its impact on the 7-day smoking point prevalence at the 30-day follow-up.

**Methods:**

US adults (n=48) were recruited via the web to use Inspired on their smartphones for 7 weeks. The object of the game was to defend a healing tree against attackers. Levels of the game were unlocked twice daily when participants self-reported the number of cigarettes they smoked since the previous entry. Completion of the levels awarded players in-game currency, which could strengthen in-game abilities. Participants received additional in-game rewards to aid gameplay by submitting either smoking self-reports only or self-reports indicating abstinence, determined through random assignment. In addition, participants completed a web-based survey at intake, week 4, week 7, and the 30-day follow-up.

**Results:**

Of the 48 participants, who had an average age of 39.8 (SD 10.7) years, 27 (56%) were female, 4 (8%) Hispanic, 37 (77%) White, and 27 (56%) employed; 26 (54%) earned <US $40,000 a year; and 14 (29%) lived in nonurban areas (eg, rural and suburban). There were no significant differences between the groups, so all outcome data were combined. Participants averaged 20.6 (SD 15.3) days of gameplay and reached a mean highest game level of 10.7 (SD 8.4), although there was a high degree of variability. Participants reported abstinence on 31.4% (SD 38.2%) of all cessation phase reports and averaged 5.4 (SD 9.8) consecutive abstinent smoke reports. For every 1 SD increase in the highest level achieved, there was a 27% increase in the percentage of abstinent samples and a 405% increase in longest continuous abstinence. At the 30-day follow-up, 23% (11/48) of the participants reported having not taken a cigarette puff in the prior 7 days and 31% (15/48) had spent at least 24 hours without smoking in the prior 14 days. On an 11-point scale, participants rated the intervention moderately favorably: if they had to do it again, they would use Inspired to help them quit (mean 6.4, SD 3.4), and Inspired was helpful in their current attempt to quit (mean 5.4*,* SD 3.6).

**Conclusions:**

These results support the acceptability of Inspired. Although high dropout rates prevent conclusions on feasibility, a subset of the participants responded favorably. Scalable and accessible video game–based smoking cessation interventions could be the key to addressing the foremost cause of preventable morbidity and mortality in the United States.

**Trial Registration:**

ClinicalTrials.gov NCT03929003; https://clinicaltrials.gov/ct2/show/NCT03929003

## Introduction

### Background

Although the prevalence of smoking has steadily declined over the past several years, cigarette smoking remains the foremost cause of preventable morbidity and mortality in the United States, accounting for approximately 480,000 deaths each year [1]. Most smokers express an interest in quitting, and approximately half of smokers attempt to quit; however, approximately 90% of quit attempts fail [2]. Evidence-based interventions such as cognitive behavioral therapy and pharmacotherapy are most likely to result in smoking cessation; however, only 31% of smokers used counseling or medication in their quit attempt, and fewer still used these strategies if they were of low socioeconomic status [2-4]. Therefore, the development of effective interventions must include efforts to optimize their accessibility and appeal to all smokers who wish to quit.

Mobile health phone apps have substantially greater reach than in-person health visits and may reach populations of smokers who wish to quit but would not otherwise access in-person smoking cessation assistance. Mobile apps hold promise in their flexibility, patient-directed treatment options, and the ability to reach individuals who are not comfortable discussing smoking cessation with a health care professional. These apps vary widely in their level of interactivity, and participant retention remains low [5,6]. Attempts to increase app use have included gamification (ie, applying game-like elements without the context of an actual game) such as goal setting, progress tracking, and earning markers such as badges [7]. Use of these features has been associated with increased motivation to quit [8], but efforts to increase subjective enjoyment of the interventions may further expand their use.

One approach that may increase subjective enjoyment while maintaining accessibility is the use of video game–based smoking cessation interventions, the main component of which consists of a playable game [9-17]. In the United States, approximately 62% of adults report playing video games, and 75% of players spend ≥3 hours playing games per week [17]. One survey conducted among smokers indicated that 75% of participants already played video games, and 65% to 67% of participants believed that a video game–based smoking cessation intervention could help them or someone they knew quit smoking [18]. With 64% of video game players in the United States reporting the use of their smartphone to play games [19], smartphones provide an accessible platform on which to develop an intervention that is scalable, acceptable, and enjoyable.

Multiple video game–based smartphone smoking cessation interventions have been developed and tested, including Inner Dragon [16], Cigbreak [17], Tobstopp [12], QuitIt [15], and Quittr [13]*.* A recent review examined other smoking intervention games, highlighting 7 studies that featured digital cessation games. These games drew on a range of theories including social cognitive theory and cognitive behavioral therapy, and they had a wide variety of game features including earning points and other rewards as well as interactive storytelling. Results were mixed, with approximately half of the studies achieving significant effects on either the smoking status or the number of cigarettes smoked, and most studies displayed high rates of dropout [14].

In an attempt to improve cessation outcomes seen in video game–based smartphone interventions, we previously developed and evaluated a prototype of a stand-alone video game called Inspired [9]. Inspired was unique because it drew from the science of contingency management, which is a powerful intervention consisting of delivering incentives when individuals meet operationally defined and objectively verified behavioral goals [20]. One common criticism of contingency management is the cost of these typically monetary incentives, so Inspired used in-game rewards whose only value was in the context of the game [21,22]. The original intention of the game was to reward users for providing objective evidence in the form of breath carbon monoxide (CO) for meeting abstinence goals, with the goal that this would function as a positive reinforcer for smoking abstinence [23]. Adult smokers recruited via the web (N=28) viewed the prototype favorably and indicated that they thought a fully developed version of the game had the potential to be fun (100%) and help them quit smoking (71%). Participants also provided feedback for improving the gameplay, such as adding variation to game levels to reduce monotony and adding narrative to engage the player.

To continue this work, a beta version of Inspired was designed and developed by an interdisciplinary team of researchers and game designers based on the prototype evaluation. The narrative of the game explained that the players were tasked with saving a village and its people from invading enemies known as “creatures of darkness.” At each level of the game, players needed to build defensive structures to protect a healing tree from the creatures. Game design elements included a core game system (ie, levels where the creatures were battled and earnings could be obtained), a metasystem (ie, where earnings could be spent to customize villages and upgrade structures), and a smoking cessation program in which players could earn in-game rewards for complying with the smoking cessation program’s objectives.

### Objectives

The original aim of the study was to examine the impact of in-game rewards dependent on objectively verified smoking abstinence on the 7-day smoking point prevalence at the 30-day follow-up. However, this objective was shifted due to technical difficulties with objective verification measures and insufficient impact of abstinence-dependent rewards (explained in the Methods section). Therefore, the purpose of this study was to evaluate the feasibility and acceptability of the beta version of Inspired as a smartphone-based smoking cessation intervention to inform future research on smoking cessation games. The main outcome remained the 7-day smoking point prevalence at the 30-day follow-up; secondary outcomes included the longest self-reported abstinence streak and percentage of abstinent self-reported submissions during the abstinence phase.

## Methods

### Ethics Approval

All procedures were reviewed and approved by the Rowan University Institutional Review Board (IRB-2014-170).

### Participants

Participants were recruited on the web through Craigslist, Facebook (Meta Platforms, Inc), Google Ads (Google LLC), and Mechanical Turk (MTurk, Amazon). Multiple advertisements with similar but varying text (eg, “test out a mobile video game designed to help you quit”), images (eg, a screenshot of gameplay), and a link to a screening survey were included. The study was submitted to ClinicalTrials.gov on September 26, 2017, and was published on the website on April 24, 2019. Participants were recruited between May 3, 2019, and July 25, 2019. The screening survey was used to obtain information on age, smoking history, desire to quit, prior experience with video games, access to mobile devices, and contact information (refer to the study by Upton et al [11] for a secondary analysis of the screening data). Inclusion criteria required participants to (1) be of legal age to purchase cigarettes in their state or jurisdiction, (2) smoke ≥10 cigarettes per day, (3) report smoking for at least 2 years, (4) report a desire to quit of ≥8 on a 10-point Likert scale, and (5) own a smartphone using the Android operating system and have an active data plan or access to Wi-Fi to connect to the internet. Exclusion criteria were (1) smoking cannabis or using other tobacco products >2 times per month, (2) having unstable medical or psychiatric conditions that may interfere with submitting accurate CO samples (eg, asthma and lactose intolerance), and (3) living with someone who smokes in the house and (4) having a career that results in excessive exposure to CO (both of which would interfere with CO sample accuracy). Respondents were also asked whether they played video games and the types of games they played. If respondents reported that they did not play games, the researcher followed up by asking whether they were interested in playing a game designed to help them quit smoking.

**Figure 1:**
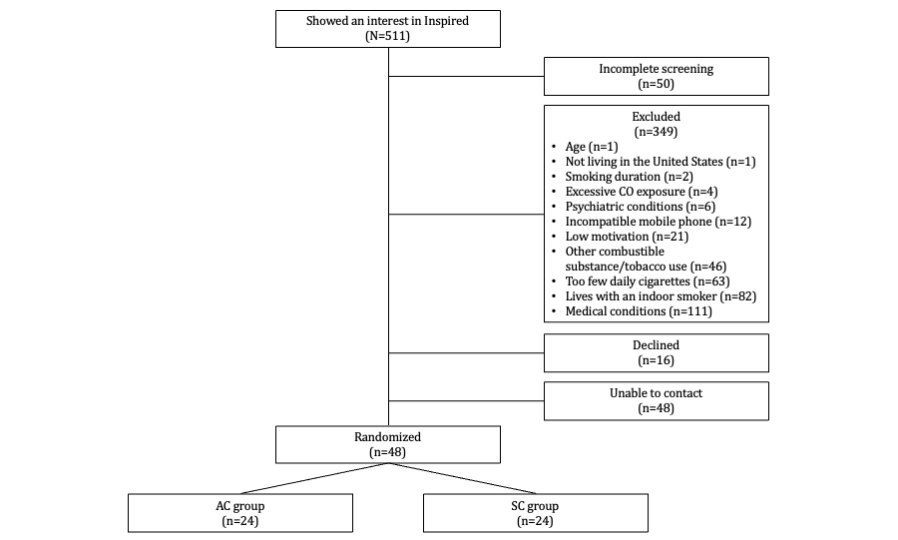
CONSORT (Consolidated Standards of Reporting Trials) diagram. AC: abstinence contingent; CO: carbon monoxide; SC: submission contingent.

A total of 511 respondents completed the screening survey, of whom 113 (22.1%) met the inclusion criteria. Respondents who met the inclusion criteria were contacted by email or phone so that study staff could explain additional information about the study and the individual could be fully informed before deciding to participate. A total of 48 participants were enrolled (see Figure 1 for a CONSORT [Consolidated Standards of Reporting Trials] diagram). Analyses were conducted in SPSS (IBM Corp) and R software (R Foundation for Statistical Computing).

The original recruitment goal was 114 participants based on a power analysis using an α level of .05 and an anticipated odds ratio of 6.68 to achieve 80% power when analyzing the effect of group on the dichotomous 7-day smoking point prevalence. However, due to considerable technical difficulties with collecting objective measures of smoking, recruitment was delayed, and a final sample of 48 participants was achieved. Initially, participants were required to submit breath CO samples to verify smoking abstinence within the last 12 hours or more using a portable breath CO detector (iCO) [24], which was attached to the participant’s phone via the headphone jack. Furthermore, to verify the identity of the participant submitting the CO sample and subsequent smoking self-report, participants were required to use facial recognition software that was built into the game. Due to technical difficulties with the vendor-supplied hardware and software, both breath CO monitoring and facial recognition technologies were removed from the procedures after a pilot trial of participants. We transitioned to relying exclusively on smoking self-reports to determine smoking status for the purposes of in-game rewards, which is the standard practice for most app-based smoking cessation trials [25]. It should be noted that the final analytical sample comprised participants who exclusively reported their smoking through self-report as opposed to CO monitoring.

Participants completed the psychosocial history questionnaire at intake, which included questions about demographic information, smoking history, and cessation history. Questions on smoking and cessation history included current tobacco use patterns (eg, the number of cigarettes smoked per day and use of other tobacco products), smoking onset (eg, age at which the first cigarette was smoked and age at which the participants started smoking daily), and quitting behavior (eg, number of previous quit attempts). Participants rated how much they wanted to quit smoking on an 11-point scale (0-10), with higher ratings representing a greater desire to quit.

Participants completed the Fagerström Test for Nicotine Dependence during intake, which consisted of 6 items. The total score summed the responses and represented the participant’s current level of cigarette dependence. A total score of 1 to 2 indicated low dependence, 3 to 4 indicated low to moderate dependence, 5 to 7 indicated moderate dependence, and ≥8 indicated high dependence [26].

The Video Game Use Survey is a 19-item measure that was administered during intake [18]. Questions assessed game playing history (eg, duration played per week) and preference for games (eg, action and adventure; strategy and puzzle) in an open-ended response format.

### Study Phases

The game was designed for 7 weeks of gameplay. Participants completed web-based survey assessments at intake, week 4, week 7, and the 30-day follow-up. Assessment content included psychosocial history, video game use, and perceptions of the intervention. Data from intake, week 7, and the 30-day follow-up were used in these analyses. Survey links were sent via email, and compensation was loaded after response completion was confirmed. Participants were mailed debit cards (CT Payer), through which compensation was delivered after completion of each battery of surveys (ie, setup: US $10, week 4: US $15, week 7: US $20, and 30-day follow-up: US $30). The compensation provided for survey completion was not connected to participant cessation efforts. Each participant was told they had the opportunity to earn a total of US $75 in compensation if all surveys were completed, regardless of whether they quit smoking.

During intake, the researcher walked each participant through downloading the Inspired app and submitting their first smoking self-report. Smoking reports, explained in more detail in the Smoking Self-Report in the Methods section, consisted of reporting the number of cigarettes smoked since the last report. Participants had the opportunity to submit 2 reports a day, waiting at least 8 hours between reports.

In the baseline phase (days 1 to 3), all participants received in-game rewards (detailed in the Gameplay section) for submitting each smoking self-report and a silver multiplier for every 3 consecutive smoking self-report submissions; however, participants were not given any smoking goals. After baseline, graduated smoking reduction and cessation goals for the remainder of the study were provided to all participants. During the tapering phase (days 4 to 7), participants were given decreasing daily target smoking caps, calculated based on the mean number of cigarettes reported during baseline, such that the final goal of the tapering phase would be 0 (eg, mean 20 cigarettes divided by 8=goal reduced by approximately 2 to 3 cigarettes per required smoke report). During the abstinence phase (weeks 2 to 4) and thinning phase (weeks 5 to 7), smoking goals were always set to 0. Initially, the schedule of rewards was programmed to end in the thinning phase. A thinning phase is common in reinforcement-based interventions and consists of gradually reducing the frequency of reinforcer delivery to reduce dependence on the reinforcer while maintaining high rates of the target behavior. This helps an individual transition to maintaining abstinence, even when the reinforcer is no longer available [27]. However, due to low rates of engagement in the thinning phase, participants received rewards at the same frequency as the abstinence phase until the end of the thinning phase.

A repeated measures group design was used. Upon enrollment, participants were randomly assigned to either a submission contingent (SC) or an abstinence contingent (AC) experimental group. All participants were given goals for smoking reduction and eventual abstinence. The SC group earned “passes” for submitting smoking self-reports on schedule, regardless of whether they reported meeting their assigned smoking reduction or abstinence goals. The AC group earned “passes” only for submitting self-reports that met their assigned smoking reduction or abstinence goals. Submissions reporting cigarette use that did not meet their smoking reduction or abstinence target were scored as a miss. For both SC and AC groups, failure to submit a self-report by the reporting deadline was scored as a miss. Thus, the only difference between the SC and AC groups was what qualified as “passing” to earn the in-game rewards. The groups were designed to compare the impact of in-game reinforcement on cessation efforts and to determine if requiring abstinence to receive rewards would be more effective than the act of reporting smoking behaviors alone, as we have done with monetary-based contingency management [28,29]. We hypothesized that the group that received in-game rewards for their abstinence would be more likely to quit smoking than the group that received in-game rewards for submitting their smoke reports only.

We compared the SC and the AC groups at intake to detect systematic group differences based on reported demographics, smoking history, cessation history, nicotine dependence, and game playing history. The 2 groups were also compared on the proportion of participants completing baseline, tapering, abstinence, and thinning phases to determine differences in dropouts.

### Gameplay

Each level in the core game began with planting a seed to grow a new healing tree. Players needed to repeatedly build and activate defensive structures to fight incoming enemies and to activate healing structures to help the tree grow. At the start of each level, players were given a bank of gems, which varied in color and shape. Players could choose to activate either a defensive or healing structure for a period of time by placing 3 gems from their bank into each structure, after which their bank would replenish with 3 randomly generated gems. Players could either place 3 random gems to power the structure for a short time or match the gem type to the “recipe” shown on the structure to power the structure for a longer period, creating a larger effect for that structure. The level was completed when the player placed an adequate number of gem sequences to sufficiently power the defense structures to push back enemies and let the healing structures fully grow their tree. Players could earn 1, 2, or 3 stars depending on how quickly the level was completed, which contributed to the player’s total score, representing the accumulated points earned across overall levels. The number of stars earned also corresponded with the amount of silver, which functioned as the in-game currency gained upon level completion.

The first 3 levels of the game served as tutorials with instructions about how to play, and they were designed to be easy to win to engage players early on. As the game continued, players were exposed to levels with progressing difficulty, which required more advanced defense structures and strategic decision-making to defeat the new enemies.

**Figure 2:**
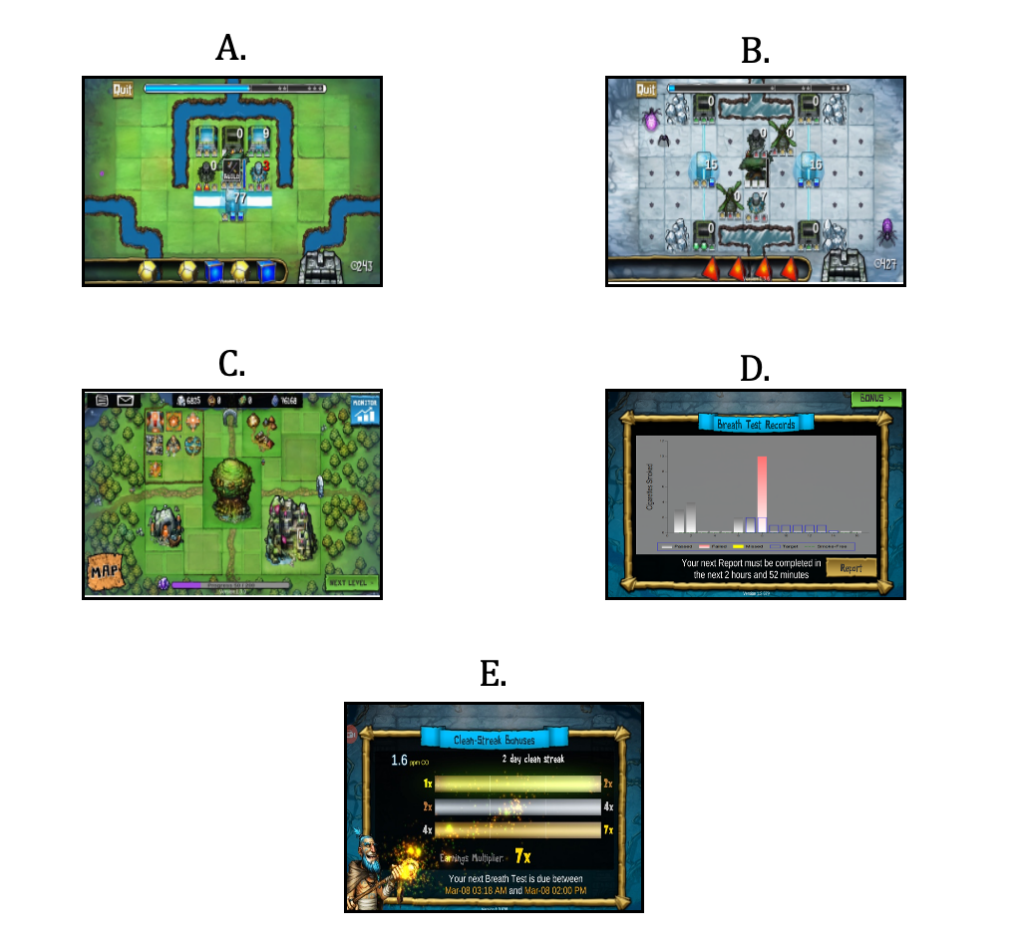
Screenshots of Inspire. (A) core game level, (B) core game level, (C) metasystem, (D) smoking report results, and (E) silver multiplier for passing smoking report (abstinence-contingent [AC] group).

In addition to playing the levels of the core game, players could access a metasystem, which was an area for activities performed outside of the core game levels. All players had a village that they could customize in the metasystem. Silver was the primary currency, earned through core level completion, that could be used to purchase cosmetic additions to customize the village and upgrade the strength of the defensive and healing structures used in the core game. Village customization was for aesthetics only and served no functional purpose, but defensive and healing structure upgrades were critical to the players’ progression through more challenging levels in the core game (see Figures 2A-2C for screenshots of each game component).

Game use outcomes included the total numerical score achieved in the game, with higher numbers indicating higher game use and achievement in completing levels. The total number of stars earned was calculated by adding the 1, 2, or 3 stars granted for the completion of each level (including replayed levels). The highest level achieved in the game and days of gameplay were also reported. Finally, the total number of defense and healing structures built in the metasystem was summed.

The Game Addiction Scale [30] assessed for indicators of gaming addiction. A total of 9 questions from the problems, conflict, and relapse subscales were rated on a 5-point Likert scale and assessed during participant intake and week 7. Scores were summed to indicate a total score of game addiction, with higher scores denoting more frequently endorsed gaming-related addictive behaviors (range 9-45). The SC and the AC groups were compared on game use (ie, the highest level, total score, total number of stars, and total number of buildings) and game addiction total scores.

### Smoking Self-Reports

As mentioned previously, players were told to submit a smoking self-report twice per day. These reports were separated by at least 8 hours and stated whether they had smoked since submitting their last report; if so, participants reported the number of cigarettes they had smoked [29]. Participants were sent push notifications that signaled when it was time to submit each report and the time by which the reports were due to avoid being recorded as “missed.”

Upon submission of each smoking self-report, the game displayed a bar graph showing the number of cigarettes reported in each prior submission so that the participants could track their smoking cessation progress over time (Figure 2D). Submission of each smoking self-report unlocked the next level in the core game system, regardless of self-reported smoking or abstinence. Therefore, players could unlock a maximum of 2 new levels per day. Participants could also replay any unlocked level as often as they liked to earn additional silver (ie, in-game currency).

Players earned in-game rewards for submitting a “passing” smoking self-report, the criteria for which depended on their group assignment (refer to the Study Phases in the Methods section). These rewards consisted of a gem prism and a silver multiplier. The gem prism was earned for every pass and could be used to select a gem color of their choice in the core game, which players could use to more easily meet the “recipes” required to power their defensive or healing structure for a longer period if their randomly generated gem bank had insufficient gems available for the recipe. Although players could match gems at random to power their structures for a shorter time, gem prisms earned through passes made fulfilling gem recipes easier, allowing for a longer structure activation and making completion of the level more likely. A silver multiplier was earned for submitting 3 consecutive passes and increased the amount of silver earned by each completed level in the core game. Multiplier values included 1×, 2×, 4×, or 7× and increased with every 3 consecutive passing smoke reports (Figure 2E). Larger quantities of silver earned through passing smoke reports could be used to more frequently upgrade the defensive and healing structures in the metasystem. This resulted in stronger structure performance, which was particularly important for later, more challenging levels.

Smoking self-report outcomes included the percentage of smoking self-reports submitted (out of the total requested reports over the course of gameplay) and the percentage of smoking self-reports indicating abstinence (out of the total submitted reports). The percentage of smoking self-reports indicating abstinence was determined for all reports throughout the gameplay and calculated for just the abstinence phase, where the goal for cigarette use was set to 0. The duration of the abstinence streak was calculated based on the highest number of consecutive reports of 0 cigarettes smoked throughout the study. The self-report submissions informed treatment dropout, which was based on the completion of each phase of the study (eg, baseline, tapering, abstinence, and thinning). Specifically, participants were scored as completing a phase if they submitted at least 1 smoking self-report in the final 2 days of the phase. The follow-up survey assessed whether participants continued to smoke once participation concluded (ie, 30 days after week 7). Participants were asked whether they had spent 24 hours without a cigarette in the previous 2 weeks and whether they had not taken a puff from a cigarette in the past 7 days. We compared the SC and the AC groups on all smoking cessation outcomes (ie, percentage of abstinent submissions during the abstinence phase and the longest duration of abstinence streak).

### Game Use on Smoking Abstinence

The proposed analyses originally planned to compare the 2 study groups, SC and AC, in the relationship between game use and smoking abstinence. This would have tested whether in-game rewards for reporting smoking abstinence were more effective in promoting cessation than rewards for reporting smoking independent of meeting abstinence goals. However, we found no systematic differences between the groups on demographics, smoking history, game use, or self-reported abstinence. In this study, earning in-game rewards (submission only or meeting abstinence goals) did not seem to impact gameplay or cessation. Therefore, the 2 groups were combined into 1 analytical group for the remaining analyses.

A total of 2 gamma generalized linear models with a log link function were created to examine the impact of total game score and the highest level achieved on both the percentage of abstinent submissions during the abstinence phase and the longest duration of abstinence. The models were conducted both with and without an interaction term between the total game score and the highest level achieved to determine the necessity of its inclusion, using the Akaike Information Criterion, Bayesian Information Criterion, and Bayes Factor as selection criteria. A generalized linear model was used to account for data heteroscedasticity, and a gamma distribution was used to accommodate a positive skew on both models.

### Cotinine Testing

Saliva cotinine testing was conducted at intake, week 2, week 4, week 7, and the 30-day follow-up to confirm smoking abstinence. Testing kits (NicAlert) [31] were mailed to all eligible participants to biologically verify self-reported tobacco use. If participants reported using cessation strategies that could elevate their cotinine levels (eg, nicotine replacement therapy or vaping nicotine), cotinine testing was not conducted. Once the kit was received, a member of the research team scheduled a video call to walk the participant through completing the test at home. After testing procedures were completed, participants submitted an image of the strip results to the researcher. A total of 2 observers independently scored all strip results and were unaware of the time point at which the test was conducted. The interobserver agreement was calculated by dividing the number of strip results agreed by the total number of strips obtained, multiplied by 100 to obtain a percentage. The interobserver agreement was 91.7% for all strip results. Participants who were eligible to submit cotinine samples received US $5 for each submission.

Verification of smoking abstinence was determined by comparing the cotinine results (positive or negative) with the most recently submitted smoking self-report on the same day the sample was collected. Participants met the correspondence criteria when both the cotinine test and the smoking self-report indicated tobacco abstinence or tobacco use. The percentage of corresponding results across all participants and time points was reported.

### Treatment Acceptability

The Treatment Acceptability Questionnaire was a 38-item survey developed to assess the perceived effectiveness of the intervention, gameplay feedback, and a comparison of how Inspired was viewed relative to other cessation strategies. Statements were rated on an 11-point scale, with 0 meaning “definitely not” to 10 meaning “yes, absolutely.” Participants were also asked to rank 10 features of the game from most to least valuable (1 being “most valuable” to 10 being “least valuable”). This questionnaire was administered at week 7.

## Results

### Participants

A total of 48 participants were randomized (n=24, 50% per group), and a summary of their characteristics can be found in Table 1. Of those participants, 27 (56%) were female, 4 (8%) were Hispanic, and 37 (77%) were White, and they had an average age of 39.8 (SD 10.7) years. A third of the sample (n=17, 35%) had never been married and most either reached high school graduation (n=15, 31%) or completed some college (n=19, 40%). Approximately half of the participants reported being employed (n=27, 56%), earning <US $40,000 a year (n=26, 54%), and renting a home (as opposed to living in a home they or someone else owned; n=26, 54%). Geographically, 14 (29%) participants lived in nonurban areas (eg, rural or suburban) [32].

**Table 1:** Participant demographics.

			Total sample (n=48)		AC^a^ group (n=24)		SC^b^ group (n=24)
Age (y), mean (SD)			39.8 (10.7)		43.0 (12.2)		36.7 (8.2)
**Sex^c^, n (%)**							
	Male	20 (42)		11 (46)		9 (38)	
	Female	27 (56)		13 (54)		14 (58)	
	Other	1 (2)		0 (0)		1 (4)	
**Ethnicity, n (%)**							
	Hispanic	4 (8)		2 (8)		2 (8)	
	Not Hispanic	44 (92)		22 (92)		22 (92)	
**Race, n (%)**							
	American Indian or Alaska Native	1 (2)		1 (4)		0 (0)	
	Black	7 (15)		3 (12)		4 (17)	
	White	37 (77)		19 (79)		18 (75)	
	Mixed race	3 (6)		1 (4)		2 (8)	
**Marital status, n (%)**							
	Currently married	15 (31)		8 (33)		7 (29)	
	Widowed	2 (4)		0 (0)		2 (8)	
	Divorced	9 (19)		4 (17)		5 (21)	
	Separated	5 (10)		4 (17)		1 (4)	
	Never married	17 (35)		8 (33)		9 (38)	
**Education, n (%)**							
	Less than high school	3 (6)		3 (12)		0 (0)	
	High school graduate or equivalent	15 (31)		6 (25)		9 (38)	
	Some college	19 (40)		10 (42)		9 (38)	
	Associate degree	8 (17)		3 (12)		5 (21)	
	Bachelor degree	3 (6)		2 (8)		1 (4)	
**Employment, n (%)**							
	Employed	27 (56)		13 (54)		14 (58)	
	Student	1 (2)		0 (0)		1 (4)	
	Not employed	20 (42)		11 (46)		9 (38)	
**Housing, n (%)**							
	Owned by the participant or household member with mortgage or loan	13 (27)		6 (25)		7 (29)	
	Owned by the participant or household member free and clear	7 (15)		3 (12)		4 (17)	
	Rented	26 (54)		14 (58)		12 (50)	
	Occupied without rent	2 (4)		1 (4)		1 (4)	
**Living in nonurban area, n (%)**							
	Yes	14 (29)		6 (25)		8 (33)	
	No	34 (71)		18 (75)		16 (67)	
**Yearly income (US $), n (%)**							
	20,000	12 (25)		6 (25)		6 (25)	
	20,000 to 39,999	14 (29)		8 (33)		6 (25)	
	40,000 to 59,999	11 (23)		6 (25)		5 (21)	
	≥60,000	11 (23)		4 (17)		7 (29)	

^a^AC: abstinence contingent.

^b^SC: submission contingent.

^c^Although gender would have been the more appropriate metric, participants were asked to indicate their sex as opposed to gender at the time of assessment.

Detailed smoking history of the participants is presented in Table 2. All participants reported currently smoking and 38% (18/48) of the participants said they also currently smoke other substances (including cannabis). On the Fagerström Test for Nicotine Dependence, participants scored an average of 6.5 (SD 1.5), indicating moderate nicotine dependence. Participants endorsed playing games (on any device, including their mobile phone) for an average of 27.1 (SD 25.4) hours per week at baseline.

**Table 2:** Smoking history.

			Total sample (n=48)		AC^a^ group (n=24)		SC^b^ group (n=24)
**Smoking other than cigarettes, n (%)**							
	Never or extremely rarely	24 (50)		12 (50)		12 (50)	
	Quit, but used to smoke	6 (12)		3 (12)		3 (12)	
	Yes, currently smoking	18 (38)		9 (38)		9 (38)	
Number of cigarettes smoked per day, mean (SD)			15.9 (8.8)		17.5 (9.7)		14.4 (7.7)
Duration of smoking (years), mean (SD)			20.2 (12.3)		23.9 (13.6)		16.5 (9.9)
Age at which the first cigarette was smoked (years), mean (SD)			16.7 (4.9)		15.4 (4.6)		17.9 (4.9)
Age of smoking regularly (years), mean (SD)			19.3 (4.9)		18.7 (4.7)		19.9 (5.1)
Desire to quit (0-10), mean (SD)			8.9 (1.3)		8.7 (1.1)		9.2 (1.4)
Number of quit attempts, mean (SD)			5.4 (5.5)		5.4 (4.3)		5.4 (6.5)
FTND^c^ total, mean (SD)			6.5 (1.5)		6.6 (1.7)		6.4 (1.3)

^a^AC: abstinence contingent.

^b^SC: submission contingent.

^c^FTND: Fagerström Test for Nicotine Dependence.

### Study Phases

**Figure 3:**
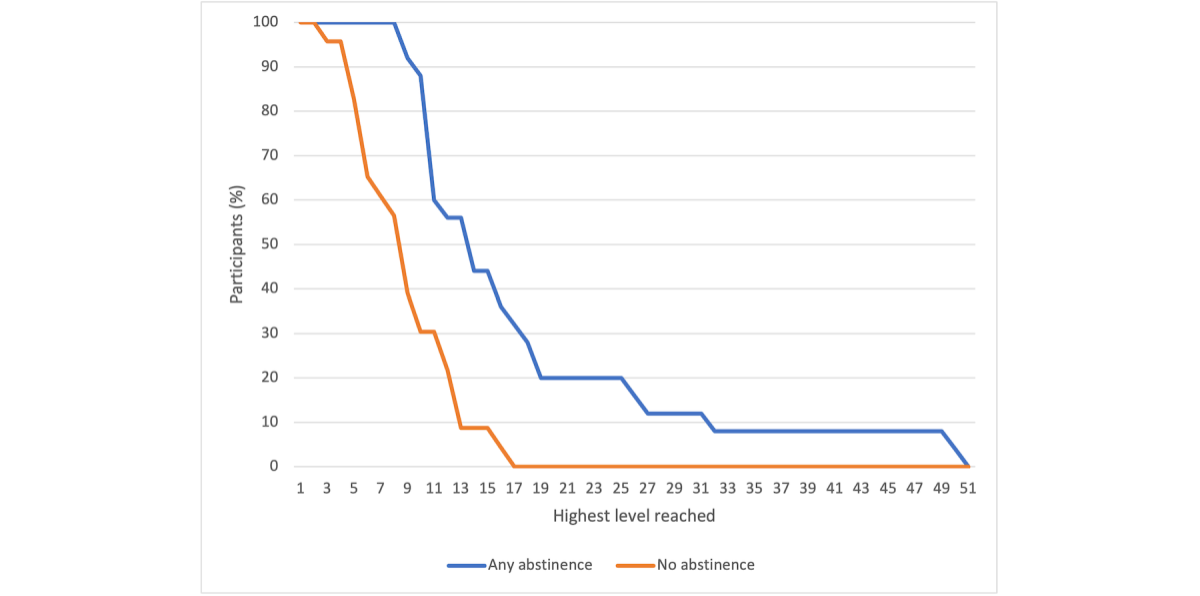
Survival curve of the highest level achieved by any abstinence reported in the abstinence phase.

Most participants completed the baseline (45/48, 94%) and tapering phases of the study (26/48, 75%). Of the total 48 participants, 20 (42%) continued to submit smoking self-reports through the end of the abstinence phase at week 4. Very few participants (n=7, 15%) submitted smoking self-reports by the end of the thinning phase (week 7). A survival curve of the highest level achieved by any abstinence reported in the abstinence phase is presented in Figure 3.

### Gameplay

The participants played the game for an average of 20.6 (SD 15.3) days over the 7 weeks of the study. They scored an average of 30,187.5 (SD 33,662.6; range 0-220,925) points and earned an average of 35.6 (SD 34.4) stars from level completions. The mean highest level reached in the game was 10.7 (SD 8.4). In the metasystem, players either built or upgraded an average of 16.4 (SD 17.6) structures. The average video game addiction score (on a scale from 9 to 45) remained stable, with scores of 14.6 (SD 4.5) at baseline versus 14.0 (SD 3.9) at week 7. Game use results are presented in Table 3.

**Table 3:** Game use.

	Total sample (n=48), mean (SD)	AC^a^ group (n=24), mean (SD)	SC^b^ group (n=24), mean (SD)	*P* value
Days of gameplay	20.6 (15.3)	18.4 (12.9)	22.8 (17.4)	.32
Highest level achieved in the game	10.7 (8.4)	8.6 (7.1)	12.8 (9.2)	.08
Total number of structures	16.4 (17.6)	14.2 (14.9)	18.6 (20.0)	.39
Total score on the game	30,287.5 (33,662.6)	22,122.9 (21,375.4)	38,452.1 (41,467.9)	.10
Total number of stars	35.6 (34.4)	26.7 (24.3)	44.5 (40.8)	.07

^a^AC: abstinence contingent.

^b^SC: submission contingent.

### Smoking Self-Reports

A detailed summary of the smoking self-report results is presented in Table 4. Of the 98 total possible report submissions over the 7 weeks of the study, participants submitted an average of 34.7 (SD 29.1; 34.7/98, 35%) reports. Participants reported smoking abstinence on 28.7 (SD 32.5; 28.7/98, 29%) reports of all 98 smoking self-reports submitted over the entirety of the intervention and on 13 (SD 38.2; 13/42, 31%) reports of all 42 reports submitted in the abstinence phase only. During the abstinence phase, 25 (52%) of the 48 participants submitted at least 1 abstinent report, meaning that nearly half of the participants did not have any abstinent reports during this phase. On average, participants reported 5.4 (SD 9.8) consecutive abstinent smoke reports.

**Table 4:** Smoke report summary.

	Total sample (n=48), mean (SD)	AC^a^ group (n=24), mean (SD)	SC^b^ group (n=24), mean (SD)	*P* value
Longest abstinence streak	5.4 (9.8)	5.3 (8.3)	5.5 (11.2)	.94
Percent smoke report submitted	34.7 (29.1)	31.5 (30.3)	37.8 (28.0)	.46
Percentage of abstinent smoke reports (out of all smoke report submissions across all phases)	28.7 (32.5)	26.9 (31.3)	30.5 (24.3)	.70
Percentage of abstinent smoke report (out of all smoke report submissions in the abstinence phase only)	31.4 (38.2)	34.1 (41.7)	28.6 (34.9)	.63

^a^AC: abstinence contingent.

^b^SC: submission contingent.

The main outcome of this examination was the 7-day smoking point prevalence at the 30-day follow-up. Of the 30 participants (across both groups) who completed the follow-up survey 30 days after the end of the intervention, 11 (37%) had not taken a puff of a cigarette in the previous 7 days and 15 (50%) said they spent at least 24 hours without smoking in the prior 2 weeks. Overall, 15 (50%) participants reported not smoking for 24 hours in the previous two weeks (AC=4, 36%; SC=11, 58%; *P*=.45) and 11 (37%) participants reported not taking a single puff from a cigarette in the previous 7-days (AC=4, 36%; SC=7, 37%; *P*=.65).

When individuals who did not respond to the survey were conservatively considered not abstinent from smoking, 11 (23%) of the 48 participants had not taken a puff of a cigarette in the previous 7 days and 15 (31%) said they spent at least 24 hours without smoking in the prior 2 weeks. Fifteen (31%) of participants reported not smoking for 24 hours in the previous 2 weeks (AC=4, 17%; SC=11, 46%; *P*=.06) and 11 (23%) reported not taking a single puff from a cigarette in the previous 7-days (AC=4, 20%; SC=7, 29%; *P*=.49).

### Game Use on Smoking Abstinence

**Figure 4:**
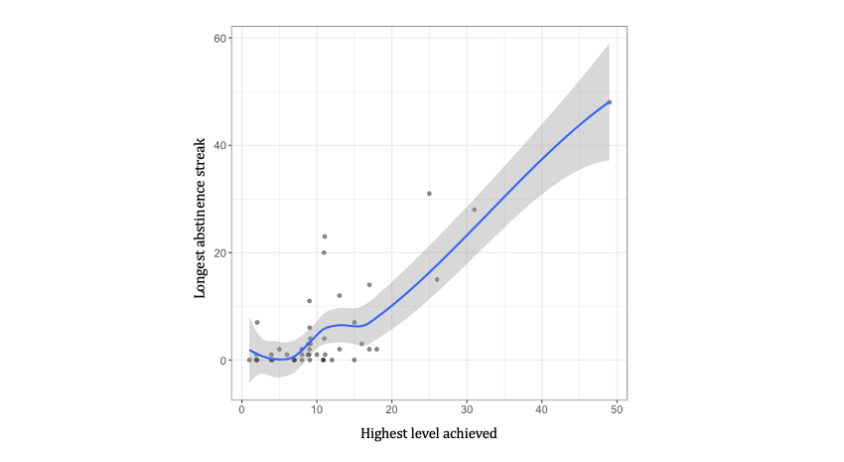
The highest level achieved by the longest abstinence streak. The longest abstinence streak is calculated by determining the maximum number of continuously reported abstinent submissions over the course of the study.

A total of 2 generalized linear models were conducted, first examining the impact of total game score and the highest level achieved on the percentage of abstinent submissions during the abstinence phase and the second examining the impact of total game score and the highest level achieved on the longest duration of continuous abstinence. Both models favored the exclusion of the interaction term, so only the individual contributions of the total game score and the highest level achieved were considered. Every 1 SD increase in total game score was associated with an 11% decrease in the percentage of abstinent submissions, whereas every 1 SD increase in the highest level achieved was associated with a 27% increase in the percentage of abstinent submissions. Similarly, an increase by 1 SD in the total score was associated with a 51% decrease in the longest abstinence streak, whereas an increase by 1 SD in the highest level achieved was associated with a 405% increase in the longest duration of continuous abstinence. In sum, the highest level achieved, but not the total game score, was positively related to both the percentage of abstinent submissions and the longest abstinence streak, with the stronger effect demonstrated between the highest level achieved and the longest duration of continuous abstinence (Figure 4).

### Cotinine Testing

Throughout the study, 20 cotinine samples were collected across all participants and at all time points. These 20 samples were compared with the number of cigarettes smoked as self-reported by the participants, 7 (35%) of whom reported tobacco abstinence. There was a 95% (19/20) correspondence between self-reported tobacco use and biologically verified cotinine results across all samples and a 100% (20/20) correspondence for the 7 abstinent reports.

### Treatment Acceptability

On an 11-point scale, ranging from 0 (“definitely not”) to 10 (“yes, absolutely”), participants rated the intervention moderately favorably. Specifically, they said that if they had to do it over again, they would use Inspired to help them quit smoking (mean 6.4, SD 3.4), that Inspired was helpful in their current attempt to quit (mean 5.3, SD 3.6), and that they would recommend Inspired to a friend (mean 6.5, SD 3.2). Participants felt the game was moderately fun to play (mean 6.7, SD 3.3), found the in-game rewards as motivating for them to submit tobacco-free smoke reports (mean 7.0, SD 3.6), and felt that the connection between in-game rewards and meeting smoking cessation goals was clear (mean 6.1, SD 3.9). More detailed results are presented in Table 5. Of the 10 game elements listed in the questionnaire, participants rated having specific-colored gems, being able to upgrade buildings to fight darkness, having waves at the end of the level that killed all remaining darkness, and being able to upgrade buildings to produce good health as the most valuable features. Apart from an open-ended “other” category, participants reported that the least valuable elements were silver to customize their village and the silver multiplier earned with consecutive tobacco-abstinent submissions.

**Table 5:** Treatment acceptability^a^ (n=30).

	Total sample (n=30), mean (SD)	AC^b^ group (n=11), mean (SD)	SC^c^ group (n=19), mean (SD)	*P* value
If you had to do it over again, would you use Inspired to help you quit smoking?	6.4 (3.4)	7.1 (3.8)	6.1 (3.3)	.44
Was Inspired helpful to you in your attempt to quit smoking?	5.4 (3.6)	5.5 (3.6)	5.3 (3.6)	.92
Do you think the Inspired game is fun to play?	6.7 (3.3)	7.3 (3.8)	6.4 (3.0)	.47
Would you recommend Inspired to a friend?	6.5 (3.2)	7.5 (3.1)	6.0 (3.2)	.22
I felt motivated to submit clean samples in order to obtain cessation points^d^	7.0 (3.6)	7.6 (3.2)	6.6 (3.9)	.45
It was clear to me how I could use the rewards I earned from meeting my goals	6.1 (3.9)	6.4 (4.3)	6.0 (3.8)	.81

^a^Scored on an 11-point scale, with 0 meaning “definitely not” to 10 meaning “yes, absolutely.”

^b^AC: abstinence contingent.

^c^SC: submission contingent.

^d^Although “abstinent” samples would be a more appropriate way to refer to these smoke reports, participants were asked to indicate their motivation to submit “clean” samples at the time of assessment.

## Discussion

### Principal Findings

The study results supported the preliminary treatment acceptability for the use of the Inspired mobile game as a smoking cessation intervention. Treatment dropout was high, and both game engagement and tobacco abstinence were highly variable, decreasing feasibility. Half of the participants (25/48, 52%) submitted at least 1 abstinent report during the abstinence phase, and game scores had a large range, suggesting that some participants may have been more receptive to the intervention than others. The main outcome, the 7-day smoking point prevalence at the 30-day follow-up, found that one-quarter (11/48, 23%) of all participants had not taken a puff of a cigarette in the prior week and a third (15/48, 31%) had spent at least 24 hours without a cigarette in the prior 2 weeks. However, it is important to note that during testing, we discovered that the game was imbalanced, and there were remaining bugs (discussed more later in the Limitations section), all of which likely prohibited game progression and led to premature dropout. These bugs likely made the in-game rewards earned through submitting smoke reports less valuable and, therefore, less likely to function as a reinforcer to facilitate smoking abstinence. Overall, participants played an average of almost 11 levels over 20 days of gameplay, and their video game addiction scores remained steady. One-third (31.4%, SD 38.2%) of submissions during the abstinence phase indicated tobacco abstinence, and participants averaged 5.4 (SD 9.8) submissions of continuous abstinence.

Of note, the AC and SC groups did not differ on gameplay and smoking cessation. It is possible that the SC group erroneously believed that they would earn in-game rewards through meeting the smoking goal requirement rather than submission of smoking reports alone. It is also possible the in-game rewards (gem prisms and silver multipliers) did not function as reinforcers. Gem prisms and silver multipliers were not required for playing the game, and their utility was most apparent for later, more challenging levels, which many participants did not reach. Participants ranked the silver multiplier among the least valuable game features in the Treatment Acceptability Questionnaire, though the helpfulness of gem prisms was not directly assessed. In addition, we do not have the data on earning and using the gem prisms and silver multipliers to determine whether the participants made contact with these rewards. Participants indicated, on the Treatment Acceptability Questionnaire, that they had a reasonably clear understanding of how the rewards in the game could motivate their smoking cessation, but participants were not asked about how they earned in-game rewards, what those rewards were, and how they could be valuable for their gameplay. Therefore, it is unclear whether the game-based incentives impacted participants’ reports of smoking.

The generalized linear models found that the highest level achieved was positively associated with the percentage of abstinent submissions and the longest abstinence streak, which suggests promise for the potential efficacy of the game to support smoking abstinence. However, the total game score was not associated with reported abstinence. Participants unlocked levels through smoking self-report submissions only and not through gameplay, whereas participants increased their score by completing levels in a sequential fashion and replaying levels, which could be done independently of submitting smoking self-reports or meeting smoking abstinence goals. In addition, the total game score had a high range and a large SD, meaning that some participants scored very little, whereas some scored very high. It is possible that the highest-scoring individuals engaged with the game more due to finding it fun or challenging rather than being related to their efforts to achieve smoking abstinence.

It is also important to note that most participants (28/48, 58%) stopped submitting smoking reports by the thinning phase. This decrease in engagement aligns with the lack of continued engagement across app-based smoking cessation interventions more broadly, which continues to be a concern in the field [33]. High dropout likely contributed to the low percentage of smoking reports submitted out of the total possible reports, at around one-third (34.7%, SD 29.1%). Finding strategies to increase and maintain engagement will be critical for future video game–based smoking cessation interventions.

The 30 participants who completed the Treatment Acceptability Questionnaire at week 7 found the game to be relatively helpful, fun, and motivating to help them quit smoking. It should be noted that participants who dropped out of the study before week 7 or who otherwise did not complete the survey may have had different opinions of the treatment, particularly if those opinions contributed to their dropout. Those who did complete the survey (30/48, 63%) highly valued the more active gameplay elements (eg, colored gems and defensive buildings) as opposed to the optional village customization that was part of the metasystem.

### Limitations

These results should be considered in the context of the study’s limitations. Due to technical difficulties, CO collection could not be performed, and smoking abstinence was determined primarily from smoking self-report. It is possible that participants falsely reported abstinence for social desirability or to gain access to in-game rewards. Although not ideal, self-reporting is common in smoking cessation studies and has aligned closely with biologically verified smoking measures in prior research [25,34,35]. We were only able to collect 20 cotinine samples; however, these demonstrated a high level of agreement with self-reported tobacco use among both abstinent and nonabstinent individuals.

The difficulties with CO collection also hindered the final sample size. The power was originally calculated for a sample size of 114 as opposed to the final sample of 48, and these analyses were based on group membership as an independent variable and the 7-day smoking point prevalence as an outcome. The study was underpowered to detect group differences in the outcome. The 2 groups were also not found to systematically differ in demographics, smoking history, game use, or self-reported abstinence. These developments informed the choice to focus on feasibility and acceptability in these analyses.

In addition, as noted earlier, participants encountered a number of bugs in the gameplay because Inspired was still in an early phase of testing. Study staff worked closely to collect this information from participants and helped troubleshoot difficulties. The high level of dropout seen throughout the study may have been due to participants returning to smoking or due to frustrations with the bugs they encountered in the game. For example, we know that level 9 of the core game was unbalanced, and many participants (9/48, 19%) stopped engaging at this level because it was too difficult to complete. Limited information was collected on exact actions during gameplay, such as the number of attempts on each level, which prevented a more granular analysis. Future examinations should focus on the playability of the game and the reasons for discontinuation. Finally, the duration of follow-up was short (30 days), preventing the examination of the intervention’s impact over a longer period.

### Strengths

This study had many strengths. Participants were relatively diverse in terms of age, education, employment, and annual income. In addition, the game was designed to deliver rewards that were only valuable in the context of the game, which, if effective, could greatly reduce the costs associated with delivering interventions that typically rely on financial incentives to promote abstinence (ie, contingency management) [36]. Although Inspired was ineffective in promoting abstinence for most participants for several potential reasons already noted, if it could be improved to promote greater levels of abstinence, it could be expanded to reach many people. Scaling up a game-based intervention that could be used by thousands of users over an extended period would add little additional cost, especially when compared to monetary-based contingency management, where each successful quit attempt, as well as longer periods of sustained abstinence, increases costs [37].

A major strength of Inspired was its flexibility. Participants were able use their mobile phone to access the game at any time of day and from any location, maximizing convenience. Using participants’ existing mobile phones made this intervention scalable and sustainable, requiring no additional hardware. Although some disparities do continue to exist concerning access to smartphones, especially among lower-income populations who are also more likely to smoke, this gap has been steadily closing, and approximately 76% of people living in households making ≤US $30,000 have access to smartphones [38]. Although participants were required to submit smoke reports twice per day, they were able to incorporate them into their existing schedule and play the game when they wished. The core game was intentionally designed to take approximately 5 minutes to complete (ie, casual game), which is about the duration of a craving arc and approximates how long it might take to smoke a cigarette, serving as a competitor to smoking [39-41]. Furthermore, common barriers to in-person smoking cessation treatment include high cost, lack of access to health care settings, and lack of cessation support from health care providers, with those of low socioeconomic status and those belonging to underrepresented groups being particularly vulnerable to disparities in care [42,43]. A mobile smartphone game such as Inspired could provide a cessation intervention regardless of the health care status or the ability to pay for treatment. The flexibility of a smartphone game–based intervention could allow for low-cost access to cessation services for a wider range of individuals.

### Conclusions

Taken together, these results showed promise for Inspired as an acceptable video game–based smoking cessation intervention. Participants were highly motivated to quit but had unsuccessfully tried to do so an average of 5.4 (SD 5.5) times before enrolling in the study. Although engagement and abstinence were variable, a subset of the participants (11/48, 23%) appeared to respond well to this approach having not taken a puff of cigarette in the previous 7 days at the 30-day follow up, which may make the difference in their smoking cessation quit attempts. Inspired used the principles of contingency management, delivering incentives, the value of which only existed within the context of the game and did not require additional monetary resources to implement [44].

Although Inspired provides an exciting alternative to traditional, in-person smoking cessation interventions, much needs to be done to ensure feasibility and scalability. The bugs in the gameplay limited the potential reinforcing value of the in-game rewards. Future examinations of Inspired and other such interventions should work to ensure that the gameplay is easy to learn and enjoyable so that achieving abstinence goals results in immediate, meaningful rewards.

The incentives offered through Inspired did not have value outside of the game, but participants may have been motivated to conceal their smoking anyway. To be in alignment with the science of contingency management, objective measures of smoking abstinence are needed. The iCO portable breath CO detector and NicAlert cotinine test strips that we attempted to use in this study were flawed [24]. Other objective measures of smoking abstinence could be explored, such as Alere rapid oral cotinine tests, which we successfully used in another study, or changes in resting heart rate that can be measured from smartphone cameras or smart watches [45,46]. The limitations of this study can inform future video game–based smoking cessation interventions, helping them fulfill the promise of using contingency management as a scalable and fun way to enact behavior change. Such low-cost and accessible interventions could be the key to achieving a healthier, smoke-free life.

## Abbreviations

AC: abstinence contingent
CO: carbon monoxide
CONSORT: Consolidated Standards of Reporting Trials
SC: submission contingent
